# Modulation of Neuropathic Pain by Glial Regulation in the Insular Cortex of Rats

**DOI:** 10.3389/fnmol.2022.815945

**Published:** 2022-04-13

**Authors:** Songyeon Choi, Kyeongmin Kim, Minjee Kwon, Sun Joon Bai, Myeounghoon Cha, Bae Hwan Lee

**Affiliations:** ^1^Department of Physiology, Yonsei University College of Medicine, Seoul, South Korea; ^2^Department of Medical Science, Brain Korea 21 Project, Yonsei University College of Medicine, Seoul, South Korea; ^3^Department of Nursing, Kyungil University, Gyeongsan, South Korea; ^4^Department of Anesthesiology and Pain Medicine, Yonsei University College of Medicine, Seoul, South Korea

**Keywords:** insular cortex, neuropathic pain, astrocytes, microglia, purinergic receptor

## Abstract

The insular cortex (IC) is known to process pain information. However, analgesic effects of glial inhibition in the IC have not yet been explored. The aim of this study was to investigate pain alleviation effects after neuroglia inhibition in the IC during the early or late phase of pain development. The effects of glial inhibitors in early or late phase inhibition in neuropathic pain were characterized in astrocytes and microglia expressions in the IC of an animal model of neuropathic pain. Changes in withdrawal responses during different stages of inhibition were compared, and morphological changes in glial cells with purinergic receptor expressions were analyzed. Inhibition of glial cells had an analgesic effect that persisted even after drug withdrawal. Both GFAP and CD11b/c expressions were decreased after injection of glial inhibitors. Morphological alterations of astrocytes and microglia were observed with expression changes of purinergic receptors. These findings indicate that inhibition of neuroglia activity in the IC alleviates chronic pain, and that purinergic receptors in glial cells are closely related to chronic pain development.

## Introduction

The International Association for the Study of Pain stipulates that, “pain is an unpleasant experience associated with actual or potential tissue damages” (Nicholas et al., 2019). Physiologically, pain occurs when sensory nerve endings called nociceptors (also referred to as pain receptors) come in contact with a pain or noxious stimulus. However, chronic pain state, as an unexpected consequence of nerve or tissue damage, metabolic disease, viral infection, or malignant tumor invasion, loses its protective effect and persists for several weeks, months, or years (Jiang et al., 2020). Ultimately, chronic pain leads to harmful and maladaptive results to human bodies. These circumstances may cause neuropathic pain (NP) and are characterized by allodynia, hyperalgesia, and spontaneous pain (Zhuo, 2007). These cardinal symptoms of NP are identified by abnormal sensory awareness and pain that persists irrespective of stimulus (Jaggi and Singh, 2011). Although many drugs have been developed to manage NP, challenges still remain (Li X.Y. et al., 2016). Pain-induced abnormal signals cause plastic changes in the peripheral and central nervous system. Formation of neuroplasticity is transmission of continuous and abnormal signals from injured sites that change the function and structure of neurons (Di Paolo et al., 2021). Thus, NP is recognized to be a consequence of neuronal plasticity caused by increase in both sensitivity and excitability of peripheral nervous system, and increase in excitability and activity of nociceptive neurons in the central nervous system (Woolf and Salter, 2000; Julius and Basbaum, 2001; Penas and Navarro, 2018). Based on this concept of pathological pain development, current results on regulation of neuroplasticity have enabled control of chronic pain (Han et al., 2016; Cha et al., 2020a; Kim et al., 2020). In particular, neuronal plasticity in the brain has been studied to be continuous with the development and maintenance of NP (Ger et al., 1990; Bak et al., 2021). Therefore, it is important to understand neuronal plasticity in parts of the brain during NP.

Clinical and laboratory studies have reported critical roles of the insular cortex (IC) in pain information processing (Emmert et al., 2014; Lu et al., 2016; Kim et al., 2017). The IC is essential for regulating nociception and is associated with pain behavior. Among structural subdivisions of the IC, especially rostral agranular insular cortex (RAIC) is a significant region that interprets pain-related emotions (Jasmin et al., 2004; Lu et al., 2016). Numerous neuronal connections of the RAIC are connected with various brain areas involved in the affective feature of pain (Lu et al., 2016; Gamal-Eltrabily et al., 2020). The RAIC receives input from the medial nucleus of the thalamus, which is related to emotional elements of nociceptive processing (Lu et al., 2016). In human studies, the rostral region of the IC was activated during harmful stimulation (Coghill et al., 1999; Brooks et al., 2002). In addition, lesions in the RAIC decreased pain-related behaviors in an animal model of NP (Jung et al., 2016; Kwon et al., 2017). These results indicated that the RAIC plays a significant role in pain information processing. Furthermore, inhibition of neuroplasticity in the IC reduced pain-related behaviors in NP models (Han et al., 2015). Also, recent studies have confirmed that changes in expression factors are essential for synaptic strengthening under chronic pain conditions (Kwon et al., 2017; Liu et al., 2020). These results suggest that the IC plays an important role in pain processing. However, factors or mechanisms that enable pain control in the IC have not been fully identified.

Emerging evidence in modulating pain-related neuroplasticity suggests that non-neuronal cells play a pivotal role in regulating neural cells (Watkins and Maier, 2003). Among them, activated astrocytes and microglia were observed in the central nervous system of a traumatic nerve injury model; thus, they were associated with induction of central sensitization and pathological pain development (Del Valle et al., 2009; Mika et al., 2013; Donnelly et al., 2020). Some studies have reported that spinal astrocytes and microglia were significantly activated after nerve injury and contribute to initiation and maintenance of NP (Scholz and Woolf, 2007; Loggia et al., 2015; Miyamoto et al., 2017). In particular, spinal microglia showed a rapid response immediately after nerve injury, and this reaction returned to baseline levels within 3 weeks (Ji et al., 2019). Contrary to microglia, activated astrocytes enhanced 1 week after nerve injury and persisted for many months. Pioneering studies on analgesic effect of glial modulation have shown that activation of astrocytes and microglia can induce or control chronic pain (Ji et al., 2013; Li et al., 2019). Nevertheless, whether activation of astrocytes or microglia takes precedence at the onset of chronic pain and at which point inhibition of cellular activity is effective for pain control remain to be elucidated. Activity change in glial cells is induced by various factors, including purinergic receptors (Jesudasan et al., 2021). Among them, it has been reported that activation of P2X receptors (P2XRs) induces secretion of glutamate and gamma-aminobutyric acid; therefore, it plays a crucial role in neuroplasticity, which is related to chronic pain (Jarvis, 2010). In the relationship between P2XR and chronic pain, activated spinal P2X4R was shown to be the key element in initiation of tactile allodynia (Tsuda et al., 2003). P2X7R, as a direct regulator of glial cell activation, has been getting attention in pain studies (Schneider et al., 2017; Trang et al., 2020). Previous studies have reported that activation mechanisms that have been identified between P2X4R and P2X7R are different, and that signals after the activation of both P2XRs are crucial for pain development (Jarvis and Khakh, 2009; Tsuda et al., 2010; Bernier et al., 2018). However, the mechanism of P2XR activation, which aggravates pain signals, has not been elucidated at the level of IC in the brain.

Here, we focused on activation change in glial cells in the IC after pain development as a consequence of peripheral nerve injury, and elucidated pain-relieving effects by suppressing astrocytes and microglia in the IC. In addition, this study aimed to investigate how P2X is involved in changes in glial cell activity. Using fluorocitrate (FC, astrocyte inhibitor) and minocycline (MC, microglia inhibitor), we sought to examine the effects of glial cell inhibition in the early and late stages of pain development. We compared the changes in protein expressions and morphology of astrocytes and microglia after glial inhibition, and further extended to the expression of purinergic receptors.

## Materials and Methods

### Experimental Animals

Male Sprague-Dawley rats (200–250 g; *N* = 268, Harlan, Koatec, Pyeongtaek, South Korea) were used for the experiments. All the rats were housed under 12-h light/dark cycle-controlled conditions in Association for Assessment and Accreditation of Laboratory Animal Care (AAALAC)-approved animal facilities. All experiments were conducted in accordance with the National Institutes of Health Guidelines. All experimental procedures were approved by the Institutional Animal Care and Use Committee of Yonsei University Health System (permit no. 2017-0076).

### Experimental Design

**FIGURE 1:**
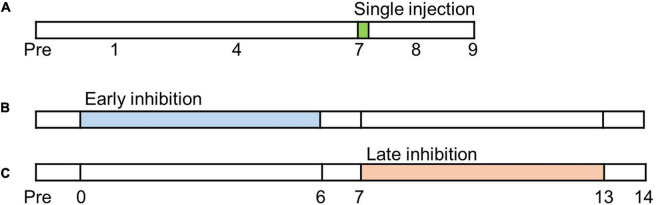
Timetable of experiments. Before neuropathic pain (NP) surgery (post-injury day, PID, 0), cannula implantations were performed. **(A)** Single injection experiment. Glial inhibitors were only injected once on PID7. **(B)** Early inhibition experiment. After NP surgery, the glial inhibitors were repetitively injected from PID0 to PID6. **(C)** Late inhibition experiment. Glial inhibitors were administered on PID7 and continued until PID13.

Mechanical allodynia is strengthened as chronic pain develops after a nerve injury (Latremoliere and Woolf, 2009). In order to determine the pain alleviating effect of FC and MC in the IC, the behavioral tests were conducted from POD 7 to POD 9 after microinjection of FC, MC or vehicle (Figure 1A). Phases of pain development can be divided into the early pain developing stage [a period immediately after nerve injury until post-injury day 7 (PID 7)] and the late pain developing stage [a period of gradual decrease in withdrawal threshold (WT), between PID 7 and PID 14] (Meacham et al., 2017; Wang et al., 2020). We designated an experiment that inhibits glial cells in the early stage of pain development as “early inhibition,” and an experiment that suppresses glial cells in the late stage of pain development as “late inhibition” (Figure 1).

### Cannula Implantation

All the rats were anesthetized with sodium pentobarbital (50 mg/kg, i.p.) and placed in a stereotaxic frame (David KOPF Instruments, Los Angeles, CA, United States). For local anesthesia, 2% lidocaine was injected into the scalp. Then, the scalp was cut to expose the bregma of the rats. Stainless steel guide cannulae (28-gauge; P1 Technologies, Roanoke, VA, United States) were bilaterally implanted into the rostral agranular IC (anterior: 1 mm from the bregma; lateral: ±4.9 mm from the midline; depth: −5.9 mm from the surface of the skull). The cannulae were fixed to the skull using stainless steel screws and acrylic cement. Dummy cannulae were inserted into guide cannulae to avert coagulation.

### Neuropathic Pain Surgery

The rats were allowed to convalesce for 3 days after the cannula implantation. The surgical procedure for nerve injury was performed following the methods described in our previous reports (Lee et al., 2000; Cha et al., 2020a,b). After recovery from the cannula implantation, an animal model of NP was prepared by ligation and cutting of sciatic nerve branches. Three branches of the left sciatic nerve were exposed, and the tibial and sural nerves were tightly ligated with 5-0 black silk and cut. The common peroneal nerve was left intact. Surgical procedures for the sham group were identical to those for the NP group but without the nerve injury procedure.

### Preparation and Administration of Glial Inhibitors

DL-Fluorocitric acid barium salt (FC, 1 nmol/μl, F9634; Sigma, St. Louis, MO, United States) and minocycline hydrochloride (MC, 10 μg/ml, M9511; Sigma) was prepared. The dose of fluorocitrate and minocycline was selected based on previous studies (Hayakawa et al., 2010; Xue et al., 2010). The vehicle solution was prepared with 0.9% NaCl solution. For microinjection, the cannula was connected to a 1-μl Hamilton syringe with a polyethylene tubing (PE-10). The drugs were slowly injected into the IC (0.5 μl/min), and the injection cannula was maintained in place for another 1 min to allow for diffusion. Even after repeated administration of fluorocitrate or minocycline to the IC of experimental animals, abnormal behaviors, weight loss, and movement disorders were not observed until the end of the experiments.

### Assessment of Mechanical Allodynia

Mechanical allodynia was assessed by measuring the WT using an electronic von Frey filament (no. 38450; UGO Basile, Varese, Italy). The rats were individually placed in an acrylic cage and habituated for 15 min. The filament was vertically applied to the medial side of the left hind paw, and force values were measured until the animals exhibited withdrawal or licking of the hind paw. The measurements were performed seven times in 2- to 3-min intervals. Then, averaged data were collected, omitting the maximum and minimum values. The von Frey test was conducted by a researcher who was blinded to the experimental groups.

### Early Inhibition

In order to evaluate the pain-relieving effect by suppressing astrocyte or microglia in the early stage, FC and MC were each injected once 1 h before the nerve injury. Glial inhibitors were administered at 0.5 μl per day. Thereafter, the nerve was injured, and the drugs were repetitively administered for 6 days at 24-h intervals. From PID 1 to PID 6, the von Frey test was performed every day, and FC or MC was injected after the completion of behavioral test. From PID 7 to PID 14, von Frey tests were performed without FC or MC administration. In order to observe morphological and molecular changes in the IC, tissues were extracted on PIDs 7 and 14. The sham group underwent sham surgery and received a vehicle solution. The vehicle group was subjected to nerve injury and was injected a vehicle solution. The FC and MC groups underwent neuropathic surgery and were injected FC and MC, respectively.

### Late Inhibition

The nerve injury rats were divided into the vehicle, FC, and MC groups. To examine the pain-relieving effects of glial inhibition in the late stage of pain development, FC and MC were administered for 7 consecutive days starting on PID 7 using the same method as early inhibition. The von Frey test was conducted 1 day before the nerve injury and on PIDs 1, 4, and 7; and the WT was reassessed daily from PID 8 to PID 14. The von Frey test was performed every day, and FC or MC was injected after the behavioral test from PID 8 to PID 14. In order to observe morphological and molecular changes in the IC, tissues were extracted on PIDs 7 and 14.

### Immunohistochemistry and Image Acquisition

On PIDs 7 and 14, the rats were deeply anesthetized with urethane and were transcardially perfused with normal saline (0.9% NaCl), followed by 4% paraformaldehyde in 0.1 M sodium phosphate buffer (PB, pH 7.4). Brains were collected and placed in 30% sucrose in phosphate-buffered saline (PBS) at 4°C for cryoprotection after 24 h post-fixation with 4% paraformaldehyde in 0.1 M PB. Samples were cryosectioned into 20-μm-thick sections on a cryostat (HM 525; Thermo Fisher Scientific, Waltham, MA, United States). The brain sections were washed three times with PBS and then blocked in 10% normal donkey serum (017-000-121; Jackson ImmunoResearch, West Grove, PA, United States) in 0.3% Triton X-100 for 1 h at room temperature (RT). The sections were incubated overnight at 4°C with the following primary antibodies: mouse anti-GFAP (1:1,000; no. 36701; Cell Signaling Technology, Danvers, MA, United States), mouse anti-CD11b/c (1:200; 550299; BD PharMingen, San Diego, CA, United States), mouse anti-NeuN (1:1,000; ab104224; Abcam, Bristol, United Kingdom), rabbit anti-P2X7 receptor (1:300; AB5246-200UL; Millipore, Burlington, MA, United States), and rabbit anti-P2X4 receptor (1:3,000; APR-002; Alomone Labs, Jerusalem, Israel). After incubation of the primary antibodies, the sections were rinsed, and a mixture of Cy3-conjugated donkey anti-mouse IgG (H + L) (1:1,000, 715-166-151; Jackson ImmunoResearch) and Alexa Fluor 488-conjugated donkey anti-rabbit IgG (H + L) (1:1,000, 711-545-152; Jackson ImmunoResearch) was applied for 2 h at RT. All the sections were mounted with a Vectashield mounting medium (Vector Laboratories, Burlingame, CA, United States). Images of astrocytes and microglia were captured using a laser scanning confocal microscope (Zeiss LSM 700; Carl Zeiss, Jena, Germany) at 20× or 40× magnitude, and were scanned in the *z*-direction at every 1 μm. Images scanned at 1 μm underwent maximum intensity projection (MIP) process using the Zen 2.3 black software (Carl Zeiss) to represent the branches and processes of astrocytes and microglia. Double-stained tissues were adjusted to constant tissue intensity and threshold in the ZEN software, and based on this, biased or bleaching signals were blocked. Then, the obtained IHC images were analyzed using the ImageJ program.^1^

### Quantification of Cell Number in the Region of Interest

To analyze the acquired images, cell morphology, and protein expression, we used contralateral RAIC (to the nerve injury) tissues. The outline of the RAIC region was confirmed with a 4×-magnitude confocal microscope. To count the number of cells in the RAIC, 20×- or 40×-magnitude fluorescence images were captured. Among them, 12 fluorescence images were randomly selected, and the number of GFAP-positive and CD11b/c-positive cells was counted.

### Quantification of Morphological Properties of Astrocytes and Microglia

Images at 40× magnification were used for morphological analysis of astrocytes and microglia. Total length, volume, number of processes, and number of nodes were obtained by Sholl analysis. The Simple Neurite Tracer (SNT) plugin^2^ was applied to the MIP images as previously described (Tavares et al., 2017). Astrocyte processes were semi-automatically traced by the software. All of the traced processes were selected, and the starting point for the analysis was set at the center of DAPI-stained nuclei. Volume was assessed by filling all paths of astrocyte reconstruction. A threshold of 0.05 yielded reproducible results for the volume analysis. The morphology of microglia was quantified by skeleton analysis and fractal analysis using the FIJI-ImageJ software as previously described (Young and Morrison, 2018). The images were converted to binary images. Then, the FIJI-ImageJ software was used to convert the binary images to skeletonized images. Furthermore, the AnalyzeSkeleton (2D/3D) plugin^3^ was applied to acquire total process length and number of end points per cell. To collect the fractal dimension of the microglia, the FracLac plugin^4^ was applied to the outlined images, which were converted from the binary images.

### Western Blot Analysis

On PIDs 7 and 14, IC (contralateral side of nerve injury) tissues were immediately extracted, frozen in liquid nitrogen, and stored at −70°C for further processing. Samples were homogenized with a lysis buffer (PRO-PREP; Intron Biotechnology, Pyeongtaek, South Korea) using an ultrasonicator and centrifuged at 15,000 rpm for 10 min. Protein samples were denatured, and equal amounts of the samples were separated and then transferred into polyvinylidene fluoride membranes (Merck Millipore, Darmstadt, Germany). The membranes were blocked with 5% bovine serum albumin solution for 1 h at RT and incubated overnight at 4°C with primary antibodies: rabbit anti-GFAP (1:10,000, ab7260; Abcam), mouse anti-CD11b/c (1:1,000, 550299; BD PharMingen), rabbit anti-P2X4 receptor (1:3,000, APR-002; Alomone Labs), rabbit anti-P2X7 receptor (1:3,000, AB5246-200UL), β-actin (1:15,000, LF-PA0207; ABFrontier, Seoul, South Korea). The membranes were then incubated with secondary anti-rabbit antibody (1:5,000, no. 7074; Cell Signaling Technology) and anti-mouse antibody (1:5,000, no. 7076; Cell Signaling Technology) for 2 h at RT. Bands were developed using the LAS system (LAS 4000; Fuji Film Co, Ltd., Tokyo, Japan) and quantified using the Multi Gauge software (Fuji Film Co., Ltd.). β-actin was used as a loading control for protein expression.

### Statistical Analyses

Statistical analyses were performed using the SPSS 20.0 software (IBM Corporation, Armonk, NY, United States). Behavioral data were analyzed by two-way analysis of variance (ANOVA) with repeated measures, followed by Bonferroni’s test for *post hoc* comparisons. Western blotting data were analyzed by one-way ANOVA followed by Bonferroni’s test for multiple comparisons. Morphometric data were analyzed by one-way ANOVA followed by Tukey’s *post hoc* test. All values are expressed as mean ± standard error of the mean (SEM). *P*-values less than 0.05 were considered statistically significant.

## Results

### Comparison of Early and Late Inhibition of Glial Cells in Chronic Pain

**FIGURE 2:**
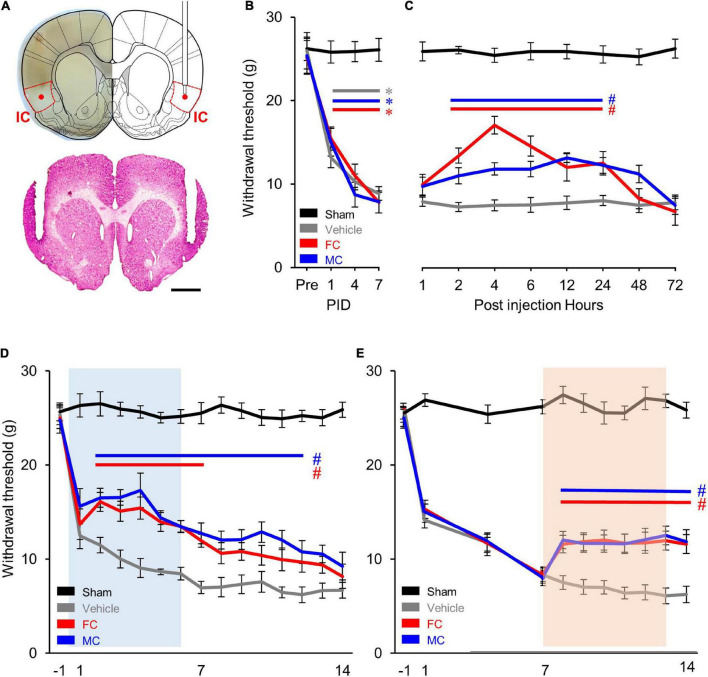
Comparison of early and late inhibition of glial cells in chronic pain. **(A)** Location of the implanted cannulae for drug injection. Dotted red lines indicate the insular cortex (IC). The guide cannulae were bilaterally implanted into the target sites, and the injection cannulae were inserted through the guide cannulae to diffuse the drugs to the target areas (red dot). **(B)** Development of mechanical allodynia after nerve injury. The vehicle- (*n* = 8) and fluorocitrate (FC)- (*n* = 8) and minocycline (MC)- (*n* = 8) treated groups that received nerve injury showed a significant decrease in mechanical withdrawal threshold on post-injury days (PIDs) 1, 4, and 7 compared to the sham group (*n* = 8). **(C)** Pain attenuation effects of single injection of FC or MC on PID 7. After drug injection, the FC-treated group (*n* = 8) showed significant pain alleviation effects for 2–24 h compared to the vehicle group (*n* = 8). Pain alleviation effects on the MC-treated group (*n* = 8) started 2 h after MC injection and was maintained for up to 24 h after injection compared to the vehicle group. **(D)** Changes in withdrawal threshold (WT) after early inhibition of astrocytes by FC or microglia by MC in the IC. After nerve injury, the WT of the vehicle group (*n* = 12) was reduced from PID 1 to PID 14 compared to the sham group (*n* = 12). The blue square on the background indicates the period of repetitive injection in early inhibition. The FC group (*n* = 12) showed significant pain-relieving effects from PID 2 to PID 7. The MC group (*n* = 12) showed significant analgesic effects from PID 2 to PID 12. **(E)** Changes in WT after late inhibition of astrocytes or microglia in the IC under chronic pain conditions. The orange square on the background indicates the period of repetitive injection in late inhibition. Both the FC (*n* = 12) and MC (*n* = 12) groups showed significant pain-relieving behaviors from PID 7 to PID 14 compared to the vehicle group (*n* = 12). Error bars are SEM; *p < 0.05 vs. Sham, ^#^*p* < 0.05 vs. Vehicle; two-way ANOVA followed by Bonferroni’s *post hoc* multiple comparison test. Each colored horizontal line indicates statistical significance.

In order to identify the effect of pain alleviation after glial cell inhibition, the cannulae were bilaterally implanted (Figure 2A) in the appropriate location. As a result, there was no difference in the WT between the four experimental groups before the NP surgery. However, the WT of the NP group (vehicle, FC, and MC) significantly decreased over time 1, 4, and 7 days after the nerve injury (each group *n* = 6). The WT of the sham group showed no difference throughout the behavioral experiment (Figure 2B). Prior to early and late inhibition trials, we determined the inhibitor’s effect in a time-dependent manner in a single injection trial. After the behavioral test on PID 7, FC or MC was injected in NP rats. Withdrawal threshold changes were measured after 1, 2, 4, 6, 12, 24, 48, and 72 h. The WT increased 2 h after FC or MC injection and was maintained for up to 24 h (Figure 2C). The FC-injected group showed significant analgesic effects for 2–24 h after the injection, and the mechanical allodynia of the MC-treated group was significantly attenuated for 2–24 h after the drug injection. However, there was no change in the vehicle group. These results showed that astrocyte and microglia inhibition result in increase in WT toward control levels.

In order to verify the pain alleviation effects with respect to the period of glial cell inhibition, we performed the early and late glial cell inhibition after nerve injury. As shown in Figure 2D, decreased WTs are observed during the first 7 days after NP surgery (PID 7). Each drug was injected into the IC every 24 h for 7 days from PID 0 to PID 6. The results of the experiment showed that the WT of the FC group decreased on PID 1. However, it rebounded on PID 2 (Figure 2D; sham, vehicle, FC, and MC; each group *n* = 12).

The WT of the FC group gradually decreased and showed significant analgesic effects until PID 7 compared to the vehicle group (Figure 2D, PID 7: sham, 25.5 ± 1.12; vehicle, 6.97 ± 0.64). From the second day of drug withdrawal (PID 8) to PID 14, the WT of the FC group did not show significant analgesic effects (Figure 2D, PID14: sham, 25.87 ± 0.8; vehicle, 6.68 ± 0.83). The MC group also showed significant pain-relieving effects compared to the vehicle group, as the WT increased from PID 2 to PID 4. Although the WT gradually decreased, it showed significant reduction in pain symptoms until PID 12 compared to the vehicle group (Figure 2D). The pain-relieving effect on the FC injection group continued during the FC injection period, whereas the pain-relieving effect on the MC-administered group continued for up to 6 days after MC discontinuation outside the MC injection period. These results indicate that suppressing astrocyte or microglial activation in the IC during the early stage of pain development alleviates the WT of the NP rats. Also, in the late inhibition of astrocytes and microglia in the IC, the WT of the NP rats rapidly decreased until PID 7 (Figure 2E; sham, vehicle, FC, and MC; each group *n* = 12, PID 7: sham, 26.33 ± 0.76; vehicle, 8.22 ± 0.71; FC, 8.17 ± 0.86; MC, 7.86 ± 0.8). Then, each drug was injected into the IC every 24 h for 7 days from PID 7 to PID 13. The WT of the FC and MC groups showed an analgesic effect compared to the vehicle group 1 day after the first injection (PID 8). A similar WT was maintained throughout the drug injection period from PID 8 to PID 14, showing a significant pain alleviation effect compared to the vehicle group. Additionally, to evaluate the sustained analgesic effect of FC and MC, the WT was measured from PID 14 to PID 21 (Figure 2E, PID 14: sham 25.35 ± 1.27; vehicle 6.06 ± 1.14; FC 12.51 ± 0.95; MC 12.01 ± 1.02; PID 21: sham 25.21 ± 0.94; vehicle 5.24 ± 1.64; FC 5.71 ± 1.37; MC 5.58 ± 1.64). As a result, the WT of the FC- and MC-injected groups started to decrease on PID15 and subsequently showed no pain alleviation effects.

### Morphometric Alterations of Astrocytes and Microglia in Early Inhibition

**FIGURE 3:**
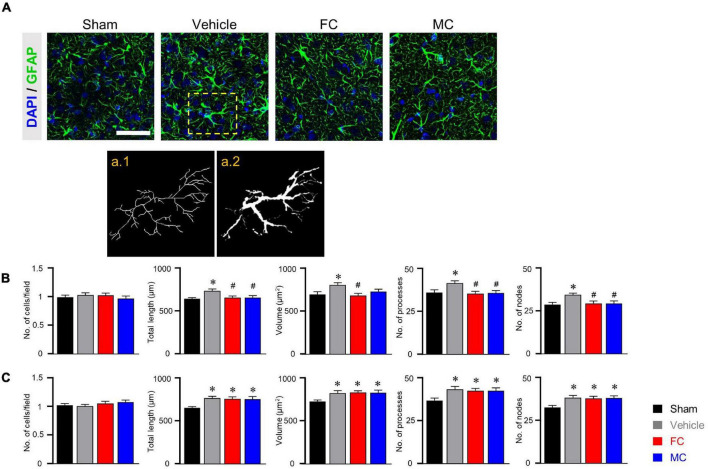
Morphometric alterations of astrocytes after early inhibition. **(A)** Representative immunofluorescence images of GFAP-positive astrocytes with cellular nuclear marker DAPI in the IC on PID 7. Scale bar, 50 μm. Increased complexity of astrocyte morphology was seen in the vehicle group compared to the sham-operated group (a.1 and a.2 represent the transformed skeletal image and volume reconstruction image of astrocytes, respectively, from yellow dotted box in the vehicle group). **(B,C)** Both FC and MC interrupted the morphological activation of astrocytes in the early stage of pain development (**B:** PID 7; **C:** PID 14). Total length, volume, number of processes, and number of nodes were quantified by the Sholl analysis protocol (sham, vehicle, FC, and MC; each group *n* = 4). Error bars are SEM; *p < 0.05 vs. sham; ^#^*p* < 0.05 vs. vehicle; one-way ANOVA followed by Tukey’s *post hoc* multiple comparison test.

Immunofluorescence staining was performed to identify morphological changes in astrocytes and microglia in the IC during the pain development stage. After the early inhibition of astrocytes and microglia on PID 7, GFAP-positive astrocytes in the vehicle group appeared to have thicker primary branches and longer processes than those in the sham group (Figure 3A). A morphometric analysis of the astrocytes was performed on PID 7 and PID 14. The results showed no differences between groups in the number of cells in the IC on PID 7 (Figure 3B, sham, vehicle, FC, and MC; each group *n* = 4; sham, 1 ± 0; vehicle 1.03 ± 0.03; FC 1.02 ± 0.04; MC 0.97 ± 0.04). However, astrocytes in the vehicle group had significantly longer processes, larger volume, increased number of processes, and more nodes in the IC than those in the sham group (Figure 3B; total length: 641.21 ± 16.41; vehicle 735.72 ± 21.46; volume: sham 695.58 ± 30.55; vehicle 805.91 ± 26.98; number of processes: sham 36 ± 1.58; vehicle 41.5 ± 1.31; number of nodes: sham 28.83 ± 1.19; vehicle 34.5 ± 0.98). In contrast, the FC group showed significant decrease in all of the measured values compared to the vehicle group (Figure 3B; total length: vehicle 735.72 ± 21.46; FC 655.19 ± 21.12; volume: vehicle 805.91 ± 26.98; FC 680.66 ± 27.77; number of processes: vehicle 41.5 ± 1.31; FC 35.41 ± 1.35; number of nodes: vehicle 34.5 ± 0.98; FC 29.5 ± 1.41). In the MC group, total length, number of processes, and number of nodes, except for volume, were significantly decreased compared to the vehicle group (Figure 3B; total length: vehicle 735.72 ± 21.46; 656.52 ± 22.89; volume: vehicle 805.91 ± 26.98; MC 729.5 ± 26.87; number of processes: vehicle 41.5 ± 1.31; MC 35.66 ± 1.52; number of nodes: vehicle, 34.5 ± 0.98; MC 29.41 ± 1.52). However, morphometric differences among the vehicle, FC, and MC groups disappeared on PID 14 (Figure 3C; number of cells: sham 1 ± 0; vehicle, 1.01 ± 0.02; FC 1.05 ± 0.03; MC 1.07 ± 0.03; total length: sham 657.73 ± 14.2; vehicle 768.95 ± 22.66; FC 762.03 ± 23.98; MC 756.72 ± 32.13; volume: sham 728.91 ± 17.86; vehicle 827.66 ± 27.94; FC 832.75 ± 21.51; MC 829.25 ± 31.89; number of processes: sham 36.66 ± 1.44; vehicle 43.25 ± 1.64; FC 42.41 ± 1.33; MC 42.41 ± 1.58; number of nodes: sham 32.83 ± 1.15; vehicle 38.58 ± 1.29; FC 38 ± 1.37; MC 38.33 ± 1.33).

**FIGURE 4:**
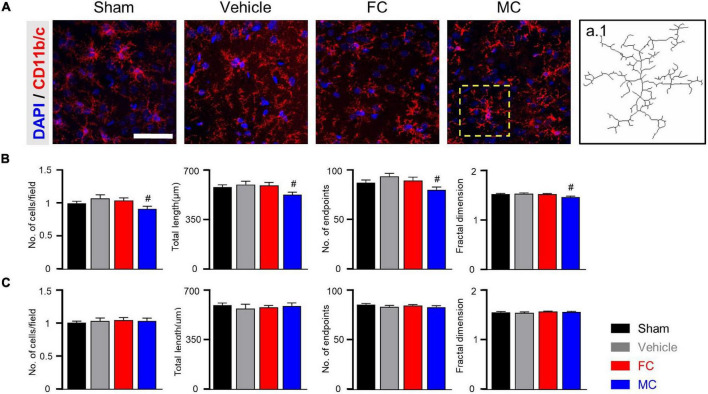
Morphological changes in microglia after early inhibition. **(A)** Representative images of CD11b/c-positive microglia with DAPI in the IC on PID 7. Decreased ramification of microglia was observed in the MC-injected group compared to the vehicle group (a.1 represents the transformed skeletal image of microglia from yellow dotted box of the MC-treated group). Scale bar, 50 μm. **(B,C)** MC inhibits morphological activation of microglia in the early stage of pain development (**B:** PID 7; **C:** PID 14). Total length, number of end points, and fractal dimension were quantified by skeleton analysis and fractal analysis (sham, vehicle, FC, and MC: each group *n* = 4). Error bars are SEM. ^#^*p* < 0.05 vs. vehicle, one-way ANOVA followed by Tukey’s *post hoc* multiple comparison test.

In CD11b/c-positive microglia, the sham, vehicle, and FC groups had similar appearances, whereas the MC group showed short branches and few processes on PID 7 (Figure 4A). The number of cells, number of endpoints, total length, and fractal dimension were significantly decreased in the MC-injected group compared to the vehicle group on PID 7. However, there was no difference in the sham, vehicle, and FC groups (Figure 4B; sham, vehicle, FC, and MC; each group *n* = 4; number of cells: sham 1 ± 0; vehicle 1.07 ± 0.04; FC 1.03 ± 0.04; MC 0.91 ± 0.04; total length: sham 575.41 ± 17.95; vehicle 595.16 ± 22.41; FC 587 ± 22.81; MC 522.75 ± 18.94; number of end points: sham 87.75 ± 2.65; vehicle 93.16 ± 2.88; FC 88.75 ± 3.5; MC 79.41 ± 3.04; fractal dimension: sham 1.51 ± 0.01; vehicle 1.52 ± 0.01; FC 1.51 ± 0.01; MC 1.45 ± 0.01). On PID 14, the differences in morphological changes disappeared (Figure 4C; sham, vehicle, FC, and MC; each group *n* = 4; number of cells: sham 1 ± 0; vehicle 1.02 ± 0.04; FC 1.04 ± 0.03; 1.03 ± 0.04; total length: sham 597.75 ± 16.37; vehicle 573.91 ± 31.86; FC 583.5 ± 13.47; MC 594.16 ± 21.1; number of end points: sham 84.91 ± 1.49; vehicle 83 ± 1.45; FC 84.25 ± 1.03; MC 82.66 ± 1.38; fractal dimension: sham 1.54 ± 0.01; vehicle 1.53 ± 0.01; FC 1.55 ± 0.01; MC 1.54 ± 0.01). These results showed that repetitive injection of FC or MC affected the glial morphology, which led to changes in pain behavior after the nerve injury.

### Morphological Changes in the Astrocytes and Microglia After Late Inhibition

**FIGURE 5:**
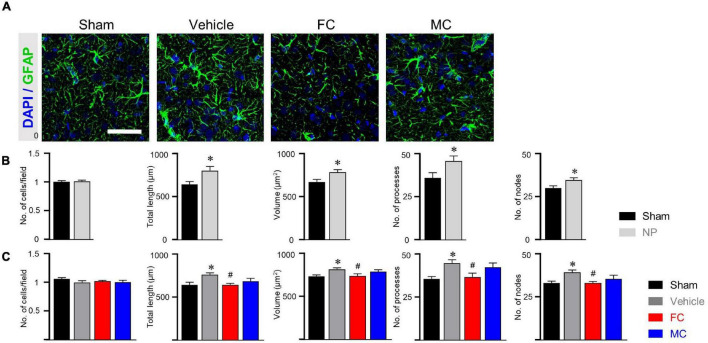
Morphological alterations in astrocytes after late inhibition. **(A)** Representative images of GFAP-positive astrocytes in the IC on PID 14. Increased complexity of astrocytes was observed in the vehicle group compared to the sham group. The FC- and MC-injected groups showed decreased morphological properties compared to the vehicle group. Scale bar, 50 μm. **(B)** Nerve injury increased the morphological complexity of astrocytes on POD 7 (sham and NP; each group *n* = 4). **(C)** Morphological complexity of astrocytes on PID 14. Total length, volume, number of processes, and number of nodes were quantified by the Sholl analysis protocol (sham, vehicle, FC, and MC; each group *n* = 4). Error bars are SEM; *p < 0.05 vs. sham; ^#^*p* < 0.05 vs. vehicle; one-way ANOVA followed by Tukey’s *post hoc* multiple comparison test.

After the late inhibition of astrocytes and microglia on PID 14, GFAP-positive astrocytes in the vehicle group appeared to have thicker primary branches and longer processes than those in the sham-operated group (Figure 5A). After the late inhibition on PID 7, the NP rats showed significantly changed astrocyte morphology except for the number of cells. Longer processes, larger volume, and increased number of processes and nodes were observed in the IC of the vehicle group (Figure 5B; sham and NP; each group *n* = 4; number of cells: sham 1 ± 0; NP 1.01 ± 0.01; total length: sham, 638.75 ± 33.18; NP 796.83 ± 48.03; volume: sham 669.91 ± 29.24; NP 782.83 ± 30.34; number of processes: sham 35.91 ± 3.1; NP 45.66 ± 2.99; number of nodes: sham 29.91 ± 1.23; NP 34.58 ± 1.27). In contrast, on PID 14, GFAP-positive astrocytes in the FC-injected group had thinner processes and primary branches than those in the vehicle group (Figure 5C; sham, vehicle, FC, and MC; each group *n* = 4). The FC-injected group showed a decrease in the morphological properties of astrocytes compared to the vehicle group. In the MC-injected group, relatively fewer branches of astrocytes were observed compared to the vehicle group. In the morphometric analyses, the number of astrocytes showed no difference in all the groups (Figure 5C; sham 1 ± 0; vehicle 1.05 ± 0.02; FC 1.02 ± 0.01; MC 1 ± 0.03). Although changes in astrocyte morphology in the MC-injected group tended to decrease, there was no statistically significant difference between the MC-injected and vehicle groups (Figure 5C; total length: sham 644.62 ± 30.03; vehicle 762.16 ± 20.94; FC 644.92 ± 17.09; MC 687.15 ± 32.63; volume: sham 738.16 ± 26.17; vehicle 800.58 ± 20.34; FC 641.91 ± 18.42; MC 744 ± 23.73; number of processes: sham 35.41 ± 1.35; vehicle 44.5 ± 1.99; FC 36.5 ± 2.21; MC 42.16 ± 2.45; number of nodes: sham 33 ± 1.05; vehicle 38.16 ± 1.41; FC 33.08 ± 0.79; MC 32.25 ± 2.29).

**FIGURE 6:**
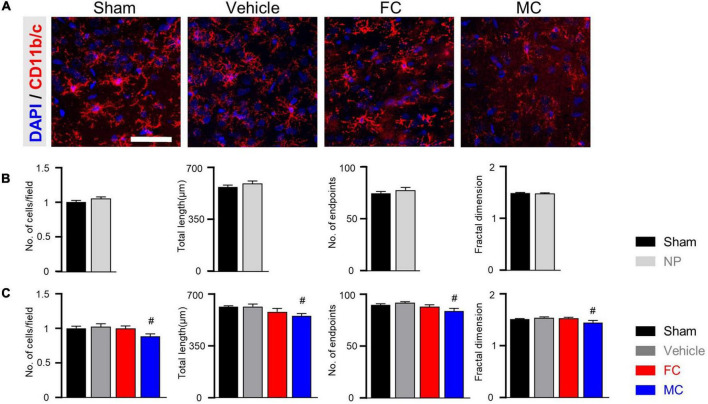
Morphometric difference in microglia after late inhibition. **(A)** Representative images of CD11b/c-positive microglia in the IC on PID 14. In the comparison of morphological changes in microglia, no difference was found except in the MC group. Scale bar, 50 μm. **(B)** Morphological complexity of microglia on PID 7. On PID 7, after late inhibition, there were no statistical differences in morphological properties among the groups (sham and NP; each group *n* = 4). **(C)** MC inhibited the morphological activation of microglia on PID 14. Total length, number of end points, and fractal dimension were quantified by skeleton analysis and fractal analysis (sham, vehicle, FC, and MC; each group *n* = 4). Scale bar, 50 μm, and error bars are SEM. ^#^*p* < 0.05 vs. vehicle, unpaired *t*-test for *post hoc* comparison and one-way ANOVA followed by Tukey’s *post hoc* multiple comparison test.

Furthermore, the CD11b/c-positive microglia of the sham, vehicle, and FC-injected groups had similar appearances, whereas the MC-injected group showed shorter branches and fewer processes (Figure 6A). The CD11b/c-positive microglia of the sham and vehicle groups had a similar appearance. There were no statistical differences in the morphological properties between the groups on PID 7 (Figure 6B; number of cells: sham 1 ± 0; NP 1.05 ± 0.02; total length: sham, 566.75 ± 13.6; NP 591.16 ± 16.53; number of end points: sham 74 ± 1.77; NP 77.25 ± 2.46; fractal dimension: sham 1.48 ± 0.01; NP 1.48 ± 0.01). In the morphological analyses, the number of cells, total length, number of end points, and fractal dimension significantly decreased in the MC group. However, there were no differences in the morphological properties of microglia among the sham, vehicle, and FC-treated groups (Figure 6C; number of cells: sham 1 ± 0; vehicle 1.02 ± 0.04; FC 0.99 ± 0.03; MC 0.88 ± 0.03; total length: sham 615 ± 7.14; vehicle 617.66 ± 16.16; FC 582 ± 22.33; MC 553.91 ± 16; number of end points: sham 89.58 ± 1.36; vehicle 91.75 ± 1.18; FC 88 ± 1.96; MC 83.83 ± 2.47; fractal dimension: sham, 1.5 ± 0.01; vehicle 1.53 ± 0.01; FC 1.52 ± 0.01; MC 1.44 ± 0.04). These results suggested that the consecutive injections of glial inhibitors, even after pain development, induced morphological changes in the astrocytes and microglia (Figures 5, 6).

### Molecular Alterations in Glial Cells After Early and Late Inhibition

**FIGURE 7:**
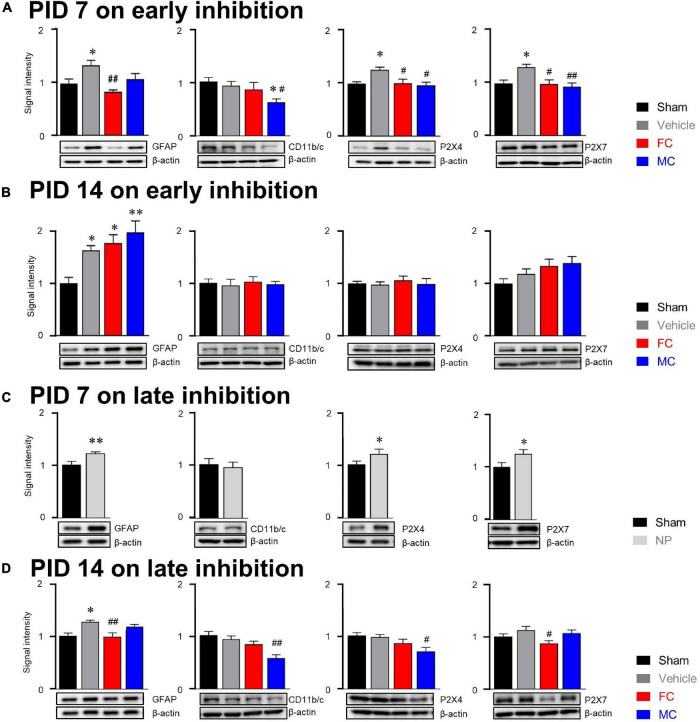
Molecular alterations in glial cells after early and late inhibition. **(A)** On PID 7, expression changes in GFAP, CD11b/c, P2X4R, and P2X7R after early inhibition of glial cells. The GFAP, CD11b/c, and purinergic receptor expressions were significantly increased in the vehicle group. The FC-treated group (*n* = 6) showed significant decrease in GFAP expression compared to the vehicle group. The MC group (*n* = 6) showed significant decrease in CD11b/c expression compared to the sham and vehicle groups (*n* = 6 each). The FC and MC groups showed significant decrease in P2X4R and P2X7R expressions compared to the vehicle group. **(B)** On PID 14 after early inhibition, expression changes in GFAP, CD11b/c, P2X4R, and P2X7R 7 days after withdrawal of inhibitors. The vehicle, FC, and MC groups showed significant increase in GFAP expression compared to the sham group (*n* = 6each). There were no significant differences in CD11b/c, P2X4R, and P2X7R expressions between the groups. **(C)** On PID 7 after late inhibition, the expression of GFAP, P2X4R, and P2X7R in the NP group was significantly increased compared to the sham group (*n* = 6). There was no significant difference in CD11b/c expression between the sham and NP groups. **(D)** Expression changes at the end of late inhibition (PID 14). The FC group showed significant decrease in GFAP and P2X7R expressions compared to the vehicle group. The MC group showed significant decrease in CD11b/c and P2X4R expressions compared to the vehicle group (sham, vehicle, FC, and MC; each group *n* = 4; error bars are SEM; *p < 0.05; ^**^p < 0.01 vs. sham; ^#^*p* < 0.05, ^##^*p* < 0.01 vs. vehicle, one-way ANOVA followed by Bonferroni’s *post hoc* multiple comparison test).

In the investigation of relationship between glial cells and purinergic receptors, our results showed that the expression of GFAP in the vehicle group was significantly increased compared to that of GFAP the sham group on PID 7 (Figure 7A; sham, vehicle, FC, and MC; each group *n* = 6: sham 1 ± 0; vehicle 1.34 ± 0.1, FC 0.83 ± 0.04; MC 1.08 ± 0.1). The expression of GFAP in the FC-injected group showed a significant decrease compared to that of the GFAP in the vehicle group. The MC-injected group showed lower expression level of GFAP than the vehicle group. However, there was no significant difference between the vehicle and MC groups. The change in expression of CD11b/c on PID 7 did not show significant difference between the sham and vehicle groups (Figure 7A; sham 1 ± 0; vehicle 0.9 ± 0.08, FC 0.85 ± 0.13; MC 0.61 ± 0.06). The expression of CD11b/c in the MC group showed a significant decrease compared to that of CD11b/c in the sham and vehicle groups. In purinergic receptor changes, P2X4R was significantly increased in the vehicle group compared to the sham group. The FC- and MC-injected groups showed a significant decrease in P2X4R expressions compared to the vehicle group (Figure 7A; sham 1 ± 0; vehicle 1.26 ± 0.04; FC 1 ± 0.07; MC 0.96 ± 0.05). The expression of P2X7R was also increased significantly in the vehicle group compared to the sham group. In contrast, P2X7R expression in the FC- and MC-treated groups showed a significant decrease compared to the vehicle group (Figure 7A; sham 1 ± 0; vehicle 1.3 ± 0.05; FC 0.98 ± 0.08; MC 0.93 ± 0.07). Interestingly, the expression of GFAP in the vehicle and FC- and MC-injected groups was significantly increased compared to the sham group after early inhibition on PID 14 (Figure 7B; sham, vehicle, FC, and MC; each group *n* = 6; sham 1 ± 0; vehicle 1.63 ± 0.09; FC 1.77 ± 0.15; MC 1.98 ± 0.21).

In addition, the expression level of GFAP in the FC- and MC-injected groups was higher than in the vehicle group. However, there were no significant changes in CD11b/c-positive microglia in all the groups on PID 14 (Figure 7B; sham 1 ± 0; vehicle 0.95 ± 0.11; FC 1.02 ± 0.09; MC 0.97 ± 0.05). The expression of P2X4R showed no significant differences among all of the groups (Figure 7B; sham 1 ± 0; vehicle 0.97 ± 0.05; FC 1.06 ± 0.07; MC 0.99 ± 0.1). The expression of P2X7R tended to increase in the vehicle group, but there were no significant differences among the groups (Figure 7B; sham 1 ± 0; vehicle 1.18 ± 0.09; FC 1.32 ± 0.13; MC 1.39 ± 0.12). These results showed that the effects of glial inhibitors disappeared on PID 14 because of withdrawal of FC and MC injections.

After late inhibition, the expression of GFAP in the NP group on PID 7 was significantly increased compared to the sham group (Figure 7C; sham and NP; each group *n* = 6; sham 1 ± 0; vehicle 1.2 ± 0.02). On the contrary, change in the expression of CD11b/c was not seen in the NP group compared to the sham group (Figure 7C; sham 1.26 ± 0.03; vehicle 0.98 ± 0.07). Expression levels of P2X4R and P2X7R were examined in the late inhibition experiment. On PID 7, both P2X4R and P2X7R expressions in the NP group were significantly increased compared to the sham group (Figure 7C; sham 1 ± 0; NP 1.19 ± 0.08). These results had a similar tendency of glia and purinergic receptor expressions, as seen in the early inhibition experiment on PID 7. On PID 14 after the late inhibition, the expression of GFAP in the vehicle group was significantly increased compared to the sham group (Figure 7D; sham, vehicle, FC, and MC; each group *n* = 6; sham, 1 ± 0; vehicle 1.26 ± 0.03; FC 0.98 ± 0.07; MC 1.16 ± 0.04). In the FC-injected group, the expression of GFAP was significantly decreased compared to the vehicle group. In the MC-injected group, there was no significant difference compared to the vehicle group. The FC-injected group showed a decrease in the expression of CD11b/c, but it was not statistically significant (Figure 7D; 1 ± 0; vehicle 0.92 ± 0.09; FC 0.86 ± 0.089; MC 0.55 ± 0.06). In the MC-treated group, CD11b/c expression was significantly reduced compared to the vehicle group.

The expression of P2X4R in the vehicle group did not show significant difference compared to the sham group (Figure 7D; sham 1 ± 0; vehicle 0.97 ± 0.04; FC 0.85 ± 0.07; MC 0.69 ± 0.07). The FC treatment downregulated the expression of P2X4R, but it was not statistically significant. Interestingly, the MC-injected group showed a significant decrease in P2X4R expression compared to the vehicle group. Increased expression of P2X7R was observed in the vehicle group; however, it was not statistically significant compared to the sham group (Figure 7D; sham 1 ± 0; vehicle 1.12 ± 0.06; FC 0.86 ± 0.05; MC 1.06 ± 0.06). The FC-injected group showed a significant reduction in P2X7R expression compared to the vehicle group. These results showed that the expression of P2X4R was significantly decreased in the MC group in which microglial activity was continuously inhibited, and that the expression of P2X7R showed a significant decrease in the FC group in which FC continuously inhibited astrocyte activity in the IC.

## Discussion

Accumulating bodies of evidence have revealed that spinal glial cells contribute to initiation and maintenance of chronic pain following peripheral nerve injury (Scholz and Woolf, 2007; Inoue and Tsuda, 2018). In this study, we identified critical roles of microglia and astrocytes in the IC from initiation to late phase of chronic pain following peripheral nerve injury. The early inhibition of glial cells attenuated allodynia and showed prolonged analgesic effect. Additionally, the suppression of glial cells in the late stage after nerve injury alleviated chronic pain. Alteration in astrocytes and microglial morphology showed reduced complexity of glial morphology after glial inhibition. P2X4R and P2X7R expressions, which tended to increase after nerve injury, were reduced after glial cell inhibition, which was consistent with the results of pain behavior. Our findings showed a close connection among glial inhibition, changes in purinergic receptors in the IC, and development of chronic pain.

Increasing evidence of glial modulation in pain studies indicates that astrocytes and microglia are closely related to transmission of pain signals (Inoue and Tsuda, 2018; Cha et al., 2020b). Earlier studies have shown that astrocytes surround the synapses of neurons and play a key role in neural signaling transmission (Singh and Abraham, 2017). Astrocytes can regulate nociceptive synaptic transmission *via* neuronal-glial and glial–glial cell interactions (Ji et al., 2019) and neuromodulators produced by microglia that can rapidly modulate synaptic plasticity, a driving force for the pathogenesis of pain after tissue and nerve injuries (Woolf and Salter, 2000; Chen et al., 2018). Although numerous studies have suggested that astrocytes and microglia are involved in pain, they are not directly involved in pain signaling. In our study, early inhibition of astrocyte activity in the IC showed pain-relieving effects and reduced GFAP expression. These results indicate that the upregulated GFAP expression implies an active state of astrocytes in the vehicle group, and that the significant reduction in GFAP expression stands for inhibited activation of it in the FC-injected group. Previous studies have demonstrated that suppression of upregulated spinal astrocyte activity was effective in attenuating chronic pain (Mannelli et al., 2014; Koller and Chakrabarty, 2020). Therefore, the pain alleviation effect in the FC-injected group indicates that the analgesic effect was induced by reduction in astrocyte activation. These results suggest that direct inhibition of astrocyte activity in the IC could alleviate chronic pain. Previous studies have confirmed significant increase in microglial activation in the spinal dorsal horn during pain condition (Lei et al., 2016; Luo et al., 2016), and have reported increase in the markers of microglial activation in several brain regions in chronic pain (Cirillo et al., 2015; Ni et al., 2016). In this study, we found that the early inhibition of glial activation by suppression of microglial activity immediately after nerve injury could delay the development of chronic pain. On the contrary, there were no differences in CD11b/c expression between the sham and vehicle groups. Moreover, we observed a downregulated expression of CD11b/c in the MC-injected rats. In previous studies, the number of activated microglia labeled with CD11b/c decreased after MC administration, and the role of microglia weakened as the number of activated microglia decreased (Fan et al., 2007; Inta et al., 2017). Interestingly, the administration of MC in the IC of the neuropathic rats also affected the transformation of astrocyte morphology. However, the administration of FC did not affect morphological changes in microglia. These results indicate that microglia affect the transformation of astrocytes and they could be influenced by the modulation of cytokines or phosphorylation of GluR1 subunit on neurons through the inactivation of microglia (Liddelow et al., 2017; Miyamoto et al., 2017).

Therefore, the pain-relieving effect on the MC group was induced by inhibition of microglial activity in the early stage. In this study, interestingly, the expression of GFAP was significantly increased in the FC- and MC-injected groups on PID 14 after drug withdrawal. The rebound in GFAP expression on PID 14 after early inhibition may be related to the disappearance of analgesic effect of FC or MC. Therefore, the rebounded expression of GFAP suggests that the activity of astrocytes, which were suppressed during the early development of chronic pain, resumed. Hence, the FC- and MC-treated groups may have higher expressions of GFAP than the vehicle group on PID 14 after early inhibition.

In this study, pain-relieving effects were also observed during the late inhibition of glial cells. Although the expression of GFAP was significantly increased after PID 7, GFAP expression was significantly decreased after seven repetitive administrations of FC in the late stage of chronic pain development (PID 14). Numerous studies have asserted that suppression of astrocyte activation could reduce chronic pain even after establishment of pain (Gwak et al., 2012; Ji et al., 2013). Late inhibition of microglial activity by injection of MC showed similar analgesic effects as the FC-injected group. The morphological changes in the MC-injected group showed a decrease in the number of activated microglia. However, the analgesic effect of MC after late inhibition disappeared a day after MC withdrawal. This result indicates that late inhibition of microglial activity has no persistent analgesic effect compared to early inhibition. A recent study, in which activity of microglia after a nerve injury was observed, confirmed the disappearance of microglial activation in the spinal cord within several days (Li Z. et al., 2016). Similar to this research, other studies have also demonstrated no analgesic effect when microglial activity is inhibited after pain development (Zhang et al., 2015; Sumitani et al., 2016). Therefore, the disappearance of prolonged analgesic effect after late inhibition of microglial activity may have been influenced by a diminished role of microglia in the late stage of pain development.

**FIGURE 8:**
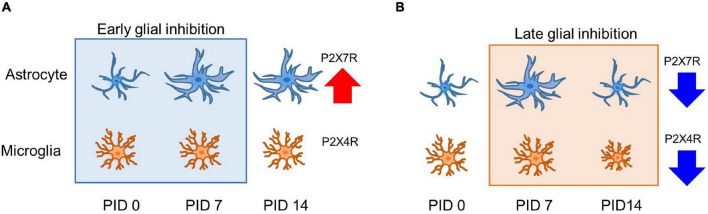
Glia and purinergic receptor changes after early and late inhibition of chronic pain. **(A)** Astrocyte and microglia change after early inhibition. Astrocytes show morphological changes with related channel expression. **(B)** Glial cell changes in late inhibition. On PID 7, microglia did not significantly change morphological shape and molecular expression after nerve injury. However, increased astrocyte shape and expression were observed on PID 7 after late inhibition, and both glial cells showed decreased expression after late inhibition on PID 14. Early and late inhibition showed different effects on the astrocytes and microglia *via* P2X4R and P2X7R. Red and the blue arrow indicates increased and reduced expressions.

In this study, both the early and late inhibition of glial cells during the pain development process showed alleviation of chronic pain establishment. Nevertheless, the change in the expressions of GFAP and CD11b/c might not fully explain the analgesic effects. The cause of these changes may explain changes in the activation of purinergic receptor expression. On PID 14, after early inhibition, the increased expression of astrocytes induced an increase in, but not a change in P2X4R. In addition, on PID 14 after late inhibition, a decrease in P2X4R by MC and a decrease in P2X7R by FC were observed, which were results of purinergic receptor-dependent glial cell expression changes. These results are summarized in Figure 8. P2X4R and P2X7R are activated under the influence of extracellular ATP (Kasuya et al., 2017). P2X4R has been known to be expressed in astrocytes and microglia, as well as in neurons (Khan et al., 2019; Illes et al., 2020). Although activation mechanisms that have been identified so far for P2X4R and P2X7R are different, signals after the activation of both P2XRs are crucial for pain development (Bernier et al., 2018). The results of our study showed that the expression of both P2X4R and P2X7R was significantly increased in the vehicle group during the early stage of pain development. In contrast, the expression levels of P2X4R and P2X7R were significantly decreased in both the FC and MC groups on PID 7 after early inhibition of glial activity. In addition, the changes in P2X4R and P2X7R expressions were consistent with the pain-relieving effect in the early stage of pain development. Together with these results, the alterations of P2X4R and P2X7R in the IC imply a close relationship between purinergic signaling and modulation of chronic pain.

In this study, there were no differences in the expression of CD11b/c and microglial morphology between the sham and vehicle groups. Moreover, the early inhibition of MC reduced the expression of P2X4R, P2X7R, and CD11b/c, whereas the late inhibition of MC controlled only P2X4R. Previous studies have confirmed that the expression of P2XRs can change without alteration of activated microglia (Inoue and Tsuda, 2018). Reduced P2X7R expression was observed in the FC-injected group. A difference in analgesic effect may occur from differently mediated purinergic receptors, resulting from interaction between astrocytes and microglia. The level of P2X7R after late inhibition showed a significant decrease in the FC-injected group. Based on the results of our western blotting, P2X4R is dominantly expressed in the microglia. These results showed that P2X4R- and P2X7R-mediated interactions between astrocytes and microglia differently affect glial cell activation in the IC during pain development. Another study reported that activation of microglia *via* P2XR was mediated by astrocyte-released ATP (Boué-Grabot and Pankratov, 2017). In addition, Barbierato et al. (2013) reported that mediators released from glial cells were significantly increased when astrocytes and microglia functioned in a cooperative manner through P2X7R-related interaction. The expression of P2X7R on PID 14 after early inhibition was similar to the GFAP expression on PID 14 after early inhibition. Based on these results, the suppression of astrocytes and microglia and changes in purinergic receptors in pain development imply that purinergic signaling in glial cells is highly related to the establishment of chronic pain. Furthermore, P2X4R is dominantly related to microglia, and P2X7R mainly corresponds to the astrocytes in the late phase of chronic pain in the IC.

Recent studies on glial cells have reported that chronic pain and glial cell activation have a close relationship not only in animal models but also in human studies (Del Valle et al., 2009; Loggia et al., 2015). Marked increase in astrocytes and microglia was confirmed in the posterior horn of the spinal cord of a patient suffering from CRPS (Del Valle et al., 2009), and a significant increase in GFAP and S100β was observed in the spinal dorsal horn of a patient with HIV and chronic pain symptoms (Loggia et al., 2015). In addition, as a result of measuring brain glia activation in a patient suffering from lower back pain using the integrated positron emission tomography/magnetic resonance imaging (PET/MRI) scanning method, an increase in glial activation in the insula was reported (Shi et al., 2012). As such, it has been reported in both animal and human studies that astrogliosis and microgliosis are closely related to development and maintenance of chronic pain, suggesting that glial cells are important factors in chronic pain. In particular, regulation of glial cell activity can be a new therapeutic approach with fewer side effects, as it is more powerful and does not directly inhibit neuronal activity. Research on glial cell activity in humans is limited due to the low-resolution imaging and it could be only at postmortem study. Therefore, it is necessary to overcome these limitations through animal studies. To obtain more definitive results, we considered the use of specific antagonists of the P2X7 and P2X4 receptors. However, no specific and potent antagonists of purinergic receptors have been developed. Therefore, detailed mechanisms of these findings could not be fully elucidated in this study, and research on the mechanism of P2X4R and P2X7R and glial activity in the rat brain of chronic pain model is still lacking. Moreover, this study did not analyze the location in which purinergic receptors are more predominantly distributed in glial cells. Therefore, more information on mechanisms of purinergic receptors and glial activity in the brain is needed to reveal the involvement of P2XR signaling in chronic pain development.

## Data Availability Statement

The original contributions presented in the study are included in the article/supplementary material, further inquiries can be directed to the corresponding author/s.

## Ethics Statement

The animal study was reviewed and approved by the Institutional Animal Care and Use Committee of Yonsei University Health System (permit no. 2017-0076).

## Author Contributions

MC and BL designed the study and supervised the research. SB coordinated and directed the project. SC and KK performed all the experiments and data analyses. KK performed the Western blot and cryosection. SC, MC, and MK wrote the manuscript. All authors contributed in critical intellectual content and approved the final version of the manuscript.

## Conflict of Interest

The authors declare that the research was conducted in the absence of any commercial or financial relationships that could be construed as a potential conflict of interest.

## Publisher’s Note

All claims expressed in this article are solely those of the authors and do not necessarily represent those of their affiliated organizations, or those of the publisher, the editors and the reviewers. Any product that may be evaluated in this article, or claim that may be made by its manufacturer, is not guaranteed or endorsed by the publisher.

## Footnotes

1
https://imagej.nih.gov/ij/

2
http://fiji.sc/Simple_Neurite_Tracer

3
http://imagej.net/AnalyzeSkeleton

4
https://imagej.nih.gov/ij/plugins/fraclac/fraclac.html
